# Predicting the Potential Distribution of *Polygala tenuifolia* Willd. under Climate Change in China

**DOI:** 10.1371/journal.pone.0163718

**Published:** 2016-09-23

**Authors:** Hongjun Jiang, Ting Liu, Lin Li, Yao Zhao, Lin Pei, Jiancheng Zhao

**Affiliations:** 1 College of Life Science, Hebei Normal University, Shijiazhuang, Hebei, China; 2 Institute of Geographical Sciences, Hebei Academy of Sciences, Shijiazhuang, Hebei, China; 3 Hebei Province Academy of Chinese Medicine Sciences, Shijiazhuang, Hebei, China; Universidad de la Republica Uruguay, URUGUAY

## Abstract

Global warming has created opportunities and challenges for the survival and development of species. Determining how climate change may impact multiple ecosystem levels and lead to various species adaptations is necessary for both biodiversity conservation and sustainable biological resource utilization. In this study, we employed Maxent to predict changes in the habitat range and altitude of *Polygala tenuifolia* Willd. under current and future climate scenarios in China. Four representative concentration pathways (RCP2.6, RCP4.5, RCP6.0, and RCP8.5) were modeled for two time periods (2050 and 2070). The model inputs included 732 presence points and nine sets of environmental variables under the current conditions and the four RCPs in 2050 and 2070. The area under the receiver-operating characteristic (ROC) curve (AUC) was used to evaluate model performance. All of the AUCs were greater than 0.80, thereby placing these models in the “very good” category. Using a jackknife analysis, the precipitation in the warmest quarter, annual mean temperature, and altitude were found to be the top three variables that affect the range of *P*. *tenuifolia*. Additionally, we found that the predicted highly suitable habitat was in reasonable agreement with its actual distribution. Furthermore, the highly suitable habitat area was slowly reduced over time.

## Introduction

Radix Polygalae, called Yuanzhi in Chinese, is a traditional Chinese herb officially listed in the medical pharmacopoeia [1,2] and is used as a mucolytic, tonic, sedative, antipsychotic, and expectorant [1,3–5]. Various medicinal components are extracted from this herb. In addition to the various saponins, xanthones, and oligosaccharides recorded in the Pharmacopoeia Commission of the People's Republic of China 2005 [2], Radix polygalae contains flavonoids, coumarins, hydroxycinnamic acid conjugates, and lignans [6]. It is the dry root of *Polygala tenuifolia* Willd. or *P*. *sibiric* L., which belong to the Polygalaceae family [1], *P*. *tenuifolia* Willd. is the most important origin plant of Radix Polygalae.

It is a perennial herb mainly distributed in Gansu, Hebei, Heilongjiang, Henan, Jiangsu, Jiangxi, Liaoning, Inner Mongolia, Ningxia, Qinghai, Shaanxi, Shanxi, and Sichuan in China, and in Mongolia, the Russian Federation, and South Korea [7]. As the understanding of Traditional Chinese Medicine has improved, sales and consumption of Radix Polygalae have been growing in last few years. But its wild resource has been getting worsen and worsen by habitat destruction and over-exploitation[8]. And this slow-growing herb has a low yield and is often harvested by dredging before seed maturation. Thus, it has been included in the List of Grade III Key State-Protected Wild Medicinal Species in China[9]. Conflict between high consumption and yield decrement has gradually forced people to exploit more wild resources and cultivate them on a large scale in recent years. However, overexploitation and promotion of blind cultivation result in loss of a valuable wild resource and a decline in the quantity and quality of Radix Polygalae. Thus, determining the optimal habitat and adaptability of wild *P*. *tenuifolia* has great implications for its protection, recovery, and utilization.

Over the period from 1880 to 2012, average global temperatures have increased by 0.85°C. The period from 1983 to 2012 was very likely the warmest 30-year period in the last 800 years [10]. A great many shifts in the distribution and abundances of species occurred in this period [11,12]. Approximately 20% of all of the world’s plant species are on the brink of extinction [13]. Thomas et al. stated that 15–37% of species in their sample of regions and taxa will be extinct based on mid-range climate-warming scenarios by 2050 [14]. Survival of many species is threatened by many issues caused by global warming, such as rising sea levels, increased rainfall, increased drought, extreme weather, and climate events [10]. Particularly, those species with high economic values face over-harvesting and environmental disruption. Although evolution of species has proven that they can adapt to the changing environment [12], enormous challenges for wild species and their ecosystems have emerged with this rapid rate of global warming. Currently, species shift, contract, expand, or fragment their range in response to the changing climate [15]. These range shifts that track suitable climatic conditions have been observed in more than 1000 species, and habitat shifts appear to be one of the most important and effective methods by which species have adapted to changes in the environment [16]. Thus, understanding the impacts of climate change on range shift of different species is important for biodiversity conservation and biological resource protection [17,18]. The increasing work on species distribution models (SDMs) has provided a new approach for predicting those changes.

SDMs were developed in the mid-1980s [19]. These models involve the comprehensive application of geographic information systems (GISs), statistics, and ecology [20–22]. In recent years, these systems have been widely used in many aspects of resource management and conservation planning [23]. By associating species occurrence with environmental variables, SDMs can model or extrapolate the occurrence of a species and yield maps of habitat suitability or a predicted species distribution [20,24]. These results can be interpreted as the probability of species presence, habitat suitability, or species richness [23,25]. These maps and distributions are imperative for biodiversity assessment [26], reserve design [27], habitat management and restoration [28], distribution modeling [29], ecological restoration [30], invasive species risk assessment [31,32], agricultural disease and insect pest forecasting [33], and impact evaluation of environmental change [14,26,34]. A number of alternative SDMs are available to predict potential species distribution, including BIOCLIM [35], BIOMAPPER [36], BRT [37], CLIMEX [38], DOMAIN [39], Favorability Function [22], GAM [40], GLM [41], GARP [42], HABITAT [43], Maxent [44], and MARS [45]. However, these models have their advantages and disadvantages. Maxent has shown higher predictive accuracy than many other methods when applied to “presence-only” species occurrence data [20,25,44,46 47].

Maxent is a general purpose machine learning algorithm [44,48]. Currently, it is the most widely used SDM [49]. Since 2006, this algorithm has been used in several studies on plants and vertebrates (e.g. birds, reptiles) [26,48,50–55]. Maxent has been used in many reports on medicinal plants. Yang et al predicted the potential distribution of *Justicia adhatoda* L. in the Himalayan foothills [56]. Remya et al. found that the habitat suitable for the distribution of *Myristica dactyloides* on the Kolli Hill in India would significantly decrease by the years 2050 and 2070 [46]. Yi et al. showed that seven variables are dominant factors in determining the suitable habitat of *Homonoia riparia* Lour [57]. Zhang et al. predicted potential suitable cultivation regions and explored the key environmental factors that affect the content of active ingredients in *Scutellaria baicalensis* Georgi in China [58]. Maxent has been demonstrated to provide the most accurate predictions using presence-only data and exhibit a better overall performance than other methods that use both presence and absence data [48,59], and it is less sensitive to overfitting [60–62].

To seek suitable habitats and evaluate the impact of climate change on *P*. *tenuifolia*, our study used Maxent to model its potential distribution based on occurrence records and environmental variables (including soil, land cover, vegetation coverage, topographical variables, and bioclimatic variables). First, dominant environmental variables were selected to build a model; second, current bioclimatic variables were used to estimate the current climatic suitable habitat; finally, potential distributions under different future climate scenarios were predicted.

## Materials and Methods

### Species records

**Fig 1:**
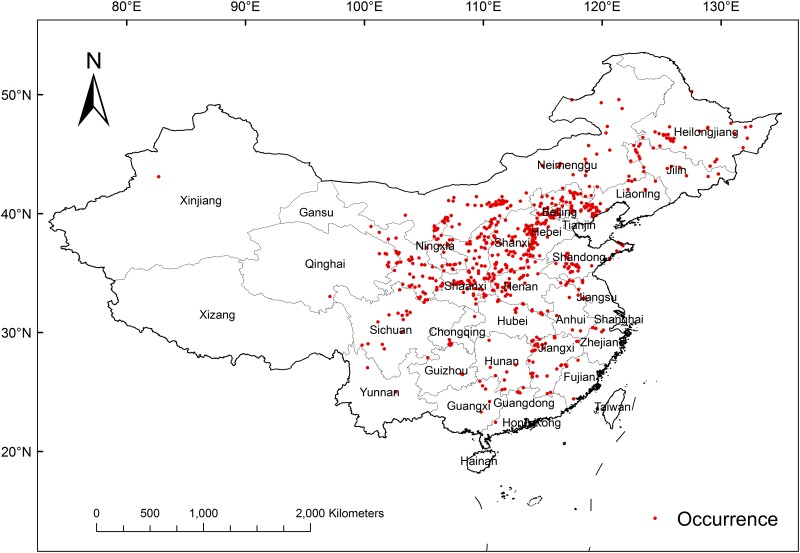
Current Distribution of *P*. *tenuifolia* Specimen Occurrences in China.

In this study, the total number of *P*. *tenuifolia* occurrences was 2332. Of these occurrence records, 2030 were collected from National Specimen Information Infrastructure (NSII; http://www.nsii.org.cn/) and Chinese Virtual Herbarium (CVH; http://www.cvh.org.cn/), 167 were collected from GBIF (http://www.gbif.org), and 135 were obtained from the fourth national survey on Chinese material medical resources. First, we georeferenced the occurrence records with detailed location information using GPS and Coordinate pick up system of Baidu Map (http://api.map.baidu.com/lbsapi/getpoint/index.html). Then, we removed 885 incomplete records as well as 657 duplicated entries. Finally, 784 accurate presence points remained. (Fig 1).

A 1 km grid [63,64] was used to reduce any negative effects that may have been caused by spatial autocorrelations in the high-density collections from populated areas [65,66]. With this grid in place, the occurrences were then filtered using SDM Tools [67]; one record was randomly picked from each grid. After filtering, 732 records remained.

#### General circulation models and environmental variables

A geographical base map of China was downloaded from DIVA-GIS (http://www.diva-gis.org). The DEM data with a 90 m spatial resolution was obtained from the CGIAR Consortium for Spatial Information (CGIAR-CSI, http://srtm.csi.cgiar.org/)[68]. Using ArcGIS 10.0 (Esri, Redlands, CA, USA), the slope and aspect were calculated.

The soil data were downloaded from the Harmonized World Soil Database (Version 1.2) [69], which was the result of a collaboration between the FAO, Chinese Academy of Sciences (ISSCAS), and several other organizations. Eighteen variables of topsoil were included in this dataset.

Global land cover and vegetation data were acquired from the International Steering Committee for Global Mapping (ISCGM, http://www.iscgm.org/gmd/).

The climate data consisted of a set of 19 bioclimatic variables that were originally derived for BIOCLIM [35], which is currently widely used in various SDMs. The current (1950–2000) climate data were obtained from the WorldClim database [70] (http://worldclim.org/), and the future (2041–2060, 2061–2080) climate data under different scenarios were obtained from Climate Change, Agriculture, and Food Security (http://www.ccafs-climate.org). The most recent climate projections were used in the Fifth Assessment IPCC report. These data were generated from general circulation models (GCMs) for four representative concentration pathways (RCP2.6, RCP4.5, RCP6, and RCP8.5). To reduce the bias in certain areas from one GCM, the multi-model ensemble (MME) average was used. This method has been shown to provide superior results to those obtained from one model [71–74], and it could maintain its essential characteristics while the number of GCMs was reduced from 25 to five [72]. Therefore, we chose five models for use in the Inter-Sectoral Impact Model Inter comparison Project (ISI-MIP) [75], including GFDL-ESM2M, HadGEM2-ES, IPSL-CM5A-LR, MIROC-SEM-CHEM, and NorESM1-M.

A total of 42 environmental variables pertaining to climate, soil, topography, land cover, and vegetation coverage were collected (S1 Table). And all environmental variables were converted into 30 arc second (~1 km) spatial resolution. High correlations among these variables decreased the accuracy of the SDM [76]; therefore, these variables were filtered to reduce this effect. The 19 bioclimatic variables were clustered into four groups that were highly correlated with the mean annual value of temperature and precipitation and their intra-annual precipitation fluctuations [77,78]. According the results in the pretest (S2 Table) and the correlations (|R| > 0.7) between variables, 20 variables were selected from the 42 variables (S2 Table) using the SDM Tools [33,67,79].

### Modeling procedure

We predicted the potential distribution of *P*. *tenuifolia* under different climatic scenarios using Maxent (http://www.cs.princeton.edu/~schapire/maxent/, version 3.3.3k, [44]). The 20 environmental variables and 732 occurrences were used as inputs for Maxent. Of these records, 25% were randomly selected to be test data, and the remainder were used as training data. The “10th percentile training presence” was chosen as the threshold rule to remove biologically irrelevant noise from the model prediction maps [64,80–82] and resulted in reliable species distributions [83].

All other parameters used the default setting. The model was replicated 10 times.

Using the parameters above, the current potential distribution of *P*. *tenuifolia* was projected, and then the future potential distributions from four different RCP scenarios (RCPs 2.6, 4.5, 6.0 and 8.5) were projected in two future periods (2050 and 2070) based on the assumptions that soil, topography, land cover, and vegetation coverage would not change under different climatic scenarios.

The area under the receiver-operating characteristic (ROC) curve (AUC) was employed in the model performance evaluation [44]. The AUC values were between 0 and 1, and higher values indicated better model performance [84]. When the AUC was below 0.5, the model performed was worse than chance. Model performance was classified as poor (0.5–0.6), fair (0.6–0.7), good (0.7–0.8), very good (0.8–0.9), or excellent (0.9–1) [84].

The jackknife results and response curves were used to evaluate the importance of each environmental variable to the distribution of *P*. *tenuifolia*. According to the presence probability, suitable regions for *P*. *tenuifolia* were divided into three levels: low suitable regions (0-T), medium suitable regions (T-0.5), and highly suitable regions (0.5–1.0). The term “T” represents the 10th percentile threshold [82]. Finally, the suitable regions and their altitudes under different climate conditions were analyzed using ArcGIS 10. Difference in the mean pixel freauency and mean altitude of highly suitable region for current and future climatic scenarios were tested using one-way ANOVAs and Tukey’s HSD comparisons in IBM SPSS Statistics 22 (IBM, Armonk, NY, USA).

## Results

### Prediction accuracy

Table 1 lists the AUC values of the Maxent predictions for the potential *P*. *tenuifolia* distributions based on environmental variables. The AUC values in all scenarios exceeded 0.8.

**Table 1:** AUC Values of Modeling *P*. *tenuifolia*’s Habitat Distribution from Four Different RCP Scenarios (RCPs 2.6, 4.5, 6.0 and 8.5) in Current and Two future Periods (2050 and 2070).

		AUC		Deviation	
		Training	Test	Training	Test
Current		0.903	0.882	0.002	0.010
2050	RCP2.6	0.902	0.877	0.002	0.008
	RCP4.5	0.905	0.876	0.003	0.009
	RCP6.0	0.906	0.882	0.001	0.004
	RCP8.5	0.905	0.882	0.002	0.006
2070	RCP2.6	0.902	0.880	0.002	0.010
	RCP4.5	0.904	0.886	0.001	0.011
	RCP6.0	0.901	0.874	0.002	0.006
	RCP8.5	0.906	0.881	0.002	0.011

### Dominant environmental variables

**Fig 2:**
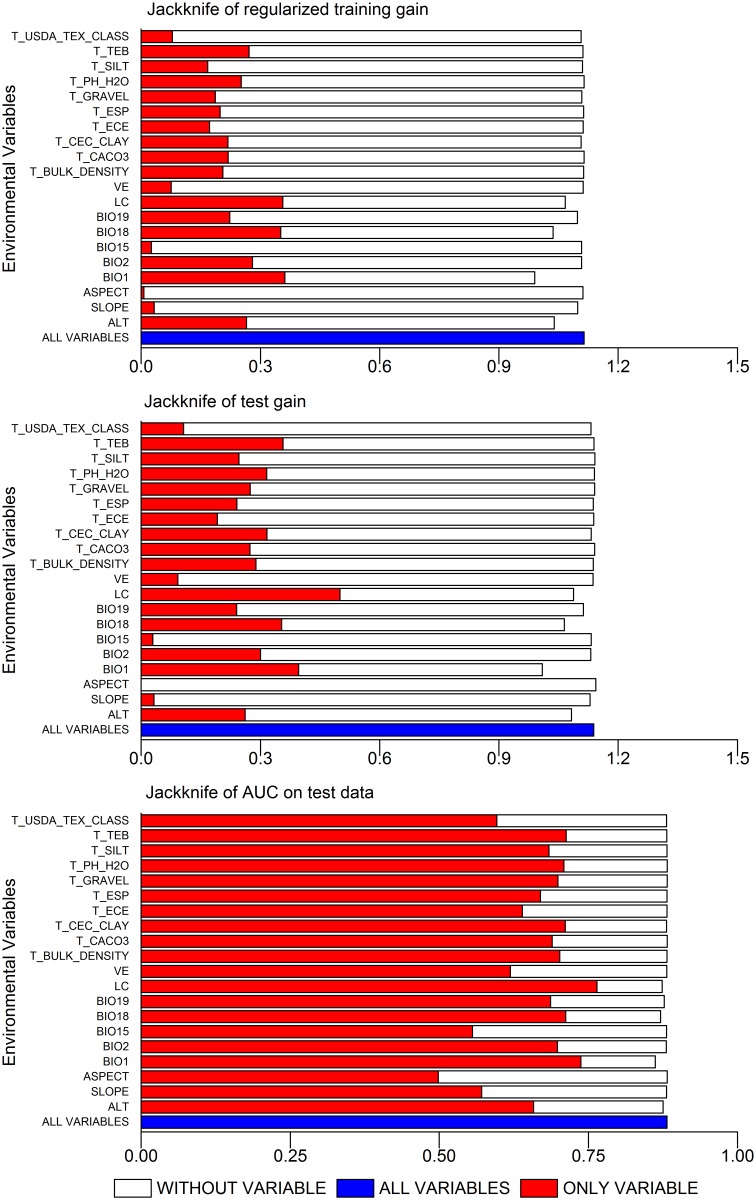
Results of the Jackknife Test of Environmental Variables’ Contribution in Modeling *P*. *tenuifolia*’s Potential Habitat Distribution. The graph shows the result of the Jackknife test of variable importance using regularized training gain, test gain and AUC on test data respectively. The red bars indicate the gain using solo environmental variable, the hollow bars indicate the gain excluding the single variable from the full model, and the blue bars indicate the gain considering all variables.

According to the results of the jackknife analyses (Fig 2) and the estimations of the relative contributions (Table 2) of environmental variables to the Maxent models under the current scenario, the top three variables that affected the distribution of *P*. *tenuifolia* were as follows: precipitation in the warmest quarter (BIO18), annual mean temperature (BIO1), and altitude (ALT). The total contribution of the three variables was 73.91%, suggesting that *P*. *tenuifolia* distributions are strongly influenced by these three environmental variables. The total contribution of five climatic variables was 61.82%. The total contribution of three topographic variables was 19.96%. However, the contribution of ten soil variables totaled 12.20%. This showed that climatic variables had a much greater impact than topography, soil, land cover, and vegetation coverage on the habitat distribution of *P*. *tenuifolia*.

**Table 2:** Relative Contributions of 20 Environmental Variables in Habitat Distribution Model of P. tenuifolia from Four Different RCP Scenarios (RCPs 2.6, 4.5, 6.0 and 8.5) in Current and Two future Periods (2050 and 2070). The abbreviations of variables could be looked up from S1 Table.

		2050				2070			
Variable Name	Current	RCP2.6	RCP4.5	RCP6.0	RCP8.5	RCP2.6	RCP4.5	RCP6.0	RCP8.5
BIO18	28.71	27.22	27.34	26.43	26.26	27.44	26.57	27.14	26.38
BIO1	28.07	31.08	29.99	27.91	28.95	29.43	27.30	28.97	25.85
ALT	17.13	15.01	15.47	17.08	16.19	17.25	16.16	17.22	17.96
LC	5.70	6.17	5.63	6.21	7.30	7.46	6.74	6.92	7.93
T_PH_H2O	2.99	3.55	3.29	3.71	4.23	3.34	4.56	3.71	3.00
BIO19	2.85	3.72	4.24	6.12	4.13	3.55	4.15	2.77	4.57
SLOPE	2.58	2.18	2.76	2.37	2.49	1.93	2.21	1.51	2.18
T_BULK_DENSITY	1.97	1.82	0.82	0.42	0.88	1.56	2.43	1.28	1.17
T_CACO3	1.94	1.92	3.65	3.25	3.38	1.78	2.03	1.54	3.61
T_TEB	1.58	1.08	0.87	1.48	0.74	0.94	0.93	1.62	0.69
BIO2	1.42	1.79	2.15	1.40	2.20	2.21	2.94	1.97	2.65
T_ECE	1.09	0.57	0.76	0.20	0.71	0.42	0.73	0.79	0.97
BIO15	0.76	0.20	0.56	0.40	0.21	0.45	0.85	1.86	0.27
T_CEC_CLAY	0.73	0.37	0.32	0.33	0.19	0.33	0.34	0.43	0.35
T_USDA_TEX_CLASS	0.58	0.90	0.41	0.48	0.43	0.47	0.60	0.56	0.57
T_GRAVEL	0.53	0.56	0.55	0.76	0.34	0.46	0.49	0.40	0.71
T_SILT	0.43	0.43	0.31	0.65	0.32	0.31	0.24	0.33	0.43
T_ESP	0.35	0.64	0.29	0.28	0.34	0.19	0.26	0.33	0.28
VE	0.32	0.38	0.36	0.29	0.40	0.32	0.29	0.49	0.28
ASPECT	0.25	0.40	0.22	0.24	0.31	0.17	0.19	0.17	0.17

### Relationship between the species distribution and the dominant environmental variables

**Fig 3:**
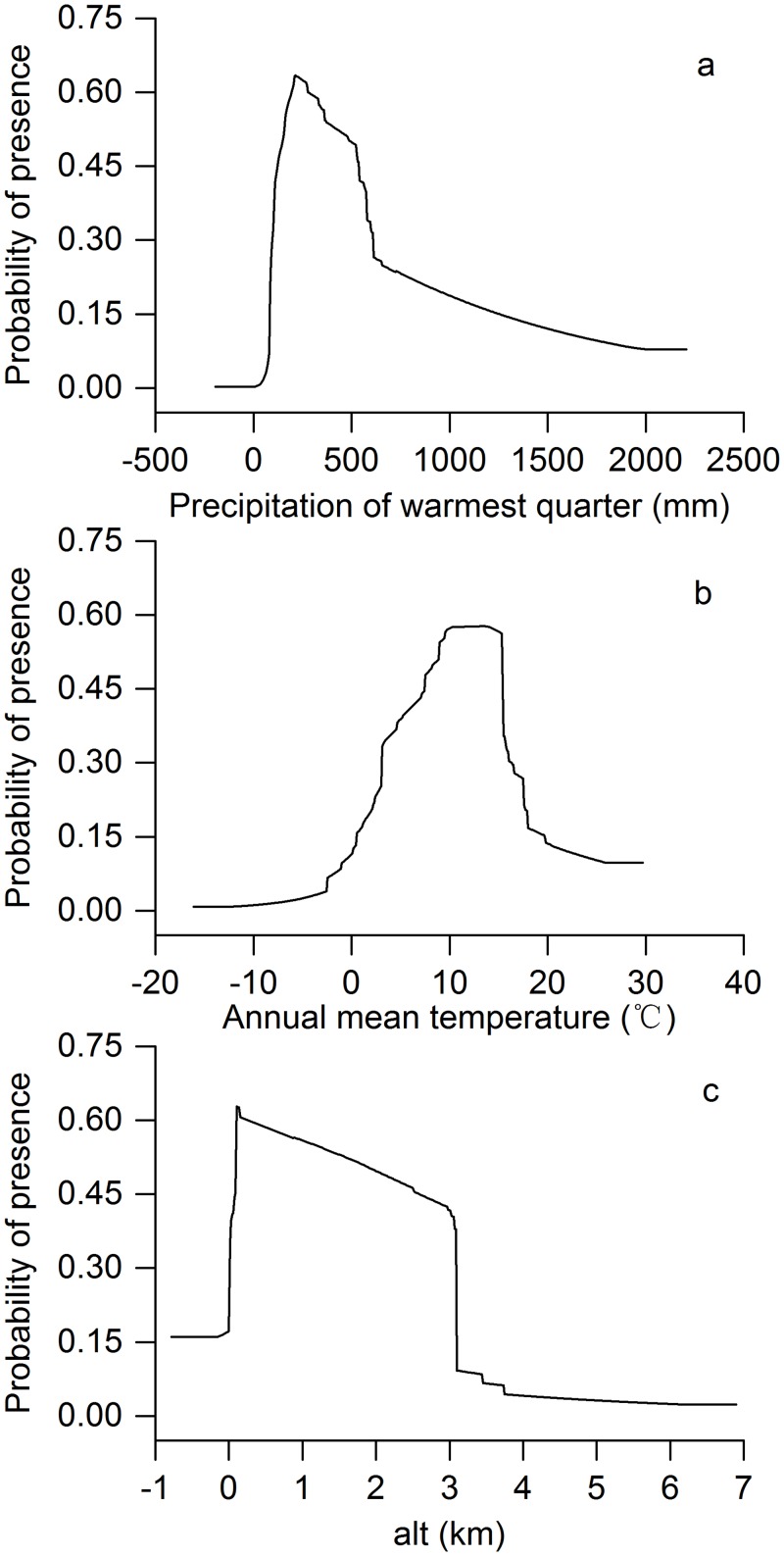
Response Curves of the Three Most Important Environmental Variables in Modeling Habitat Distribution for *P*. *tenuifolia*. (a) Precipitation in the Warmest Quarter, (b) Annual Mean Temperature and (c) Altitude.

The relationship between presence probability of *P*. tenuifolia and precipitation in the warmest quarter (Fig 3a) showed that when the precipitation was below 23 mm, the presence probability of finding *P*. *tenuifolia* was less than 5%. When the precipitation in the warmest quarter ranged between 148 mm and 512 mm, the presence probability was higher than 50%. Precipitation in the warmest quarter from 148 mm and 512 mm might be effective predictor for modeling the potential distribution of *P*. *tenuifolia*.

The annual mean temperature was another important variable that affected the distribution of *P*. *tenuifolia* (Fig 3b). When the annual mean temperature fell below -2.5°C, the presence probability of finding *P*. *tenuifolia* was less than 5%. When this temperature was in the range of 8.4°C to 15.4°C, the presence probability was higher than 50%. In other words, regions with such a range of annual temperature may be better candidates for planting *P*. *tenuifolia*.

Altitude also played an important role in forecasting the potential distribution of *P*. *tenuifolia*, which displayed a presence threshold of 3740 m (Fig 3c). The presence probability gradually declined with increasing elevation from 100 m to 3087 m.

### Potential suitable distribution areas for *P*. *tenuifolia*

**Fig 4:**
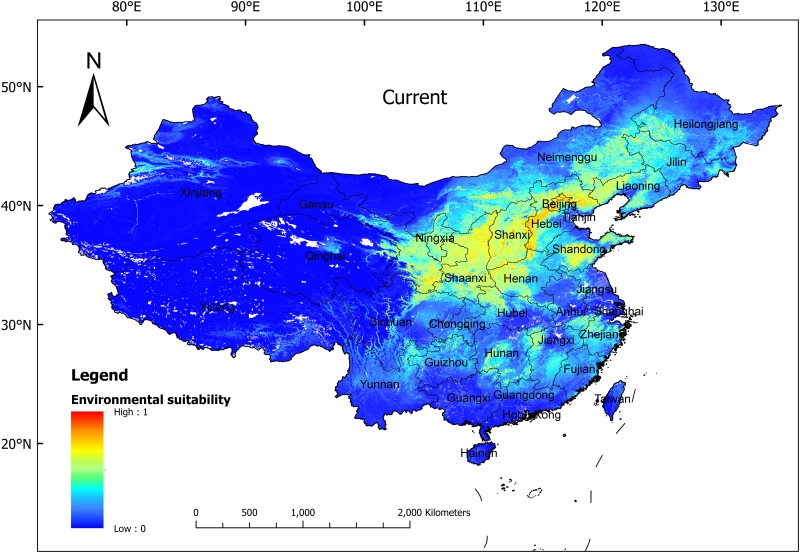
Potential Distribution Modeled by Maxent for *P*. *tenuifolia* in China under the Current Climate. Blue to red colors show the increase of presence probability. 0 indicates completely unsuitable and 1 indicates optimal.

Using ArcGIS 10.0, the potential distribution in the current situation predicted by Maxent was analyzed (Fig 4). It showed that the highly suitable regions (the presence probability was greater than or equal to 50%) for *P*. *tenuifolia* are primarily located in the north of China, including the following: West Liaoning, North and West Hebei, most of Beijing, Central and South Shanxi, the northwest corner and west of Henan, North and Central Shaanxi, East and South Gansu, Northwest Hubei, Central Shandong, and some fragmented plots in Heilongjiang, Jilin, Inner Mongolia, Ningxia, Anhui, Zhejiang, Jiangxi, Hunan, Chongqing, Sichuan, and Guizhou. The total area of this region is approximately 0.68 × 10^6^ km^2^, which accounts for 7.07% of the total land in China.

The medium suitable region was located around the edges of the highly suitable region as follows: Southwest Heilongjiang, most of Jilin, Central and West Liaoning, Northwest and Southeast Hebei, West and East Shandong, East Henan, West Hubei, South Shaanxi, Central Gansu, the east corner of Qinghai, most of Ningxia, Central and West Inner Mongolia, the northeast corner of Yunnan, North Guizhou, Central Hunan, West and Southeast Jiangxi, West Zhejiang, South Anhui, and other scattered regions. The total area of this region was approximately 1.21 × 10^6^ km^2^, which accounts for 12.62% of the total land in China. The low suitable region had the widest distribution, which included Xinjiang, Qinghai, Xizang, West Gansu, most of Sichuan, most of Yunnan, Guangxi, Guangdong, Hainan, Taiwan, West and North Inner Mongolia, and North Heilongjiang. The total area of this region was approximately 7.71 × 10^6^ km^2^, which accounts for approximately 80.31% of the total land in China.

**Fig 5:**
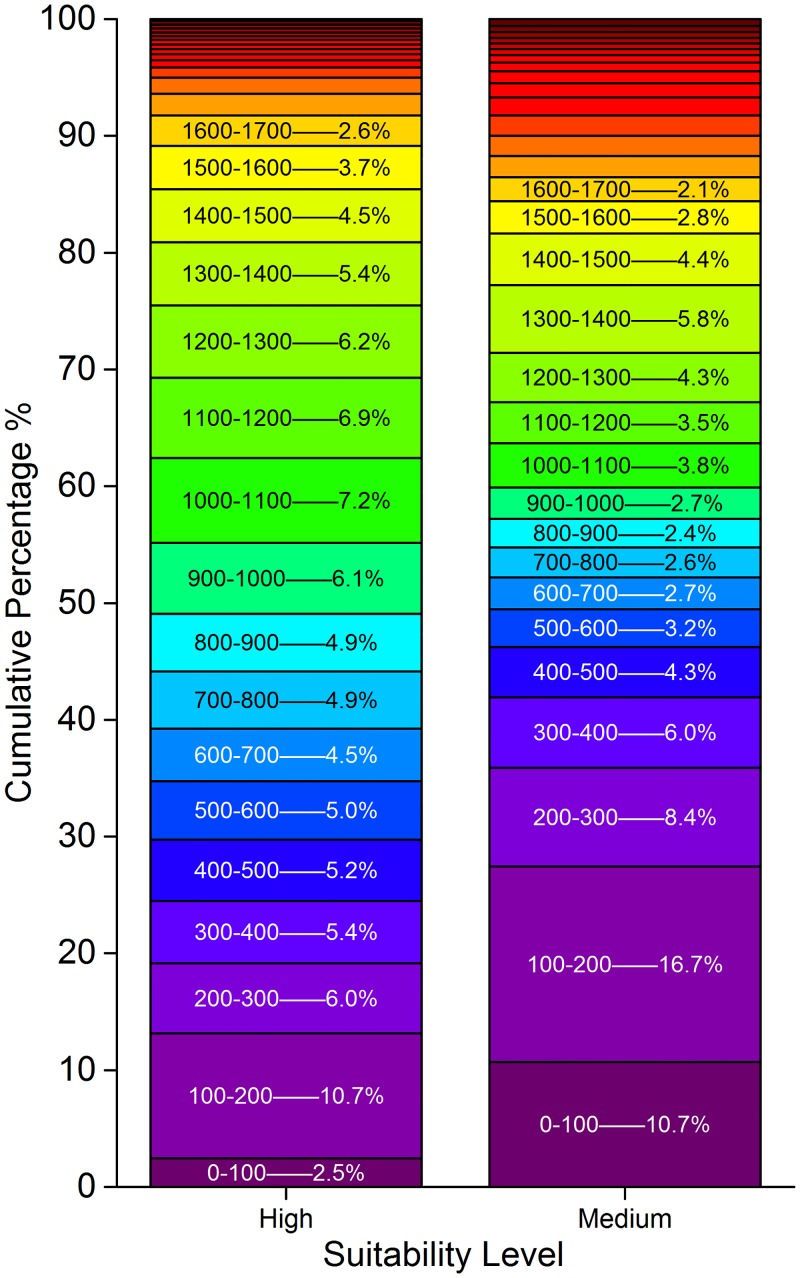
Altitudinal Pattern of High Suitable Habitat and Medium Suitable Habitat for Potential Distribution of *P*. *Tenuifolia* in the Current Scenario. The cells indicate the proportion of each hundred altitude to the whole.

Furthermore, the altitudinal pattern of the highly suitable habitat under the current situation was analyzed. Thirty percent of the highly suitable habitat was below 500 m, 70% was below 1200 m, and almost 90% was below 1700 m. The highly suitable habitat above 1700 m consisted of slightly less than 10%. In the medium suitable region, the region below 200 m accounted for nearly 30%, the region below 600 m accounted for more than 50%, and 90% of this region was not higher than 2000 m (Fig 5).

### Changes of the suitable climatic conditions according to climate warming scenarios

**Fig 6:**
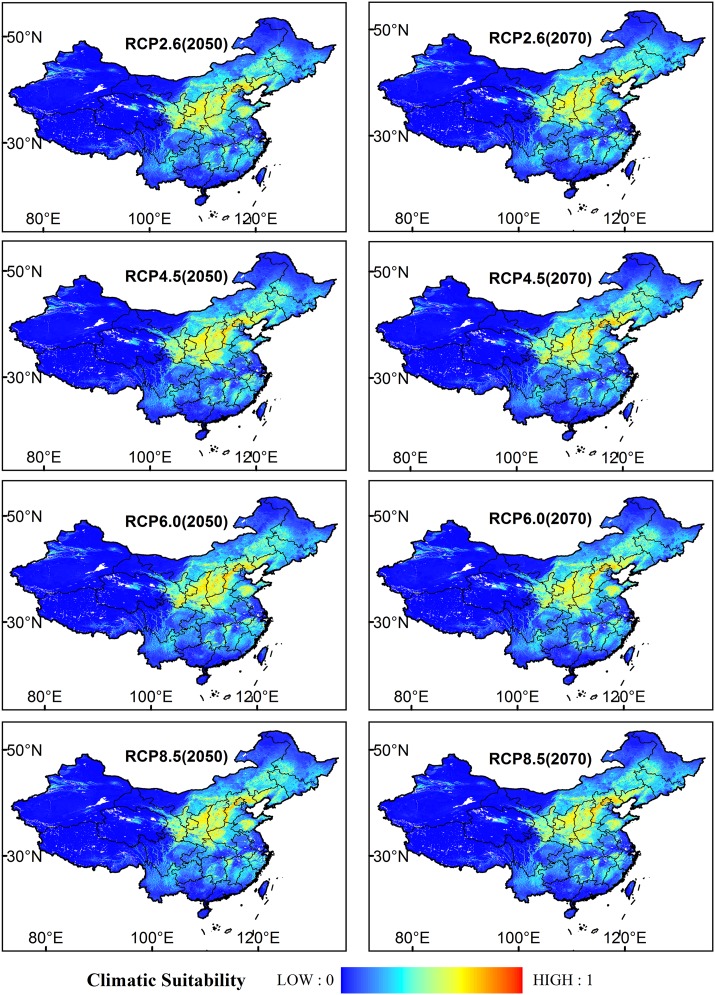
Potential Distribution of *P*. *tenuifolia* Modeled by Maxent Under the Four RCPs (RCPs 2.6, 4.5, 6.0 and 8.5) in the Two Periods (2050 and 2070). Blue to red colors indicate the presence probability of the areas from 0 to 1.

The potential distributions of *P*. *tenuifolia* under the four RCP scenarios (RCP2.6, RCP4.5, RCP6.0 and RCP8.5) in 2050 and 2070 were compared and analyzed in Fig 6.

**Fig 7:**
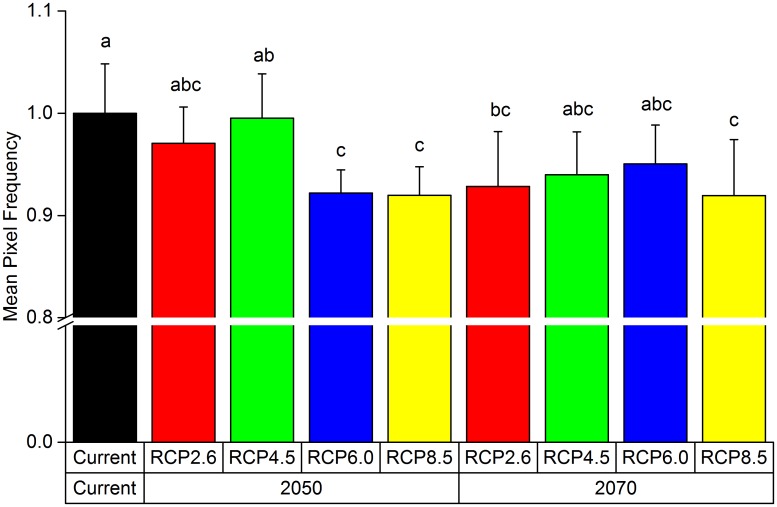
The Change of Highly Suitable Habitat Area of *P*. *tenuifolia* under the Four RCPs (RCP2.6, RCP4.5, RCP6.0 and RCP8.5) in Two Periods (2050 and 2070). The mean pixel frequency of highly suitable habitat in the current scenario was set as 1.0 and the others were expressed relative to it. Bars indicate means ± sd (n = 10 circle), and significant differences at P < 0.01 within in each group are indicated by different letters (a, b and c).

**Fig 8:**
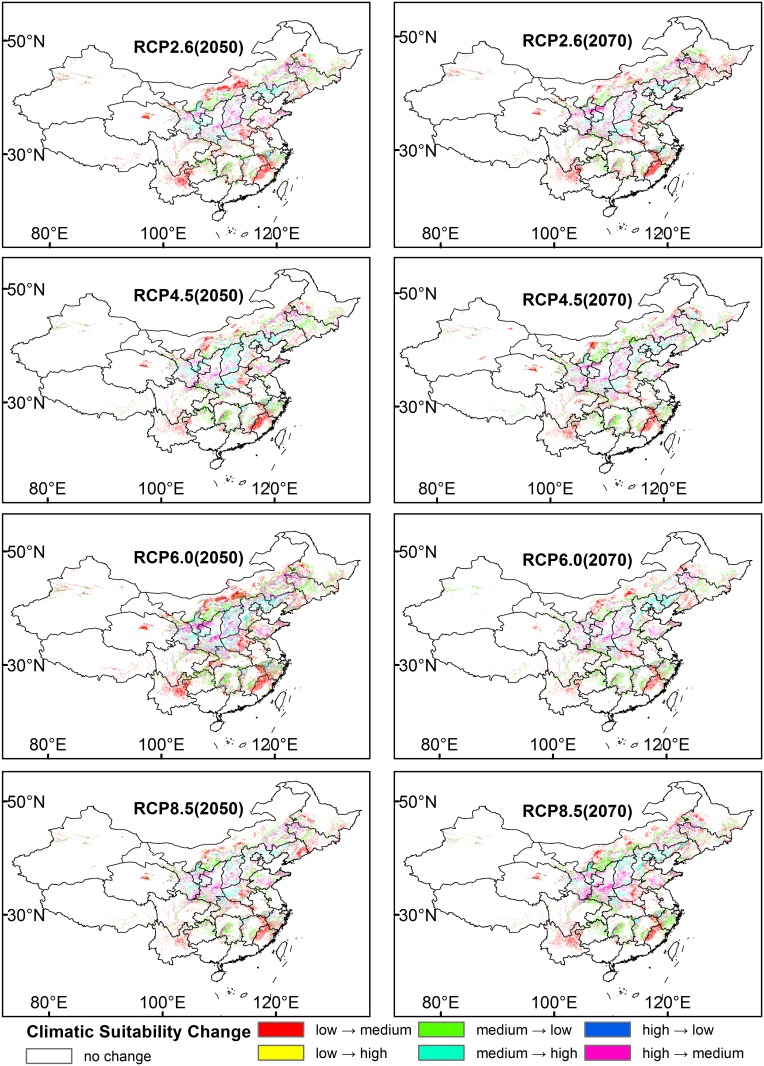
Shift in the *P*. *tenuifolia* Potential Distribution under the Four RCPs (RCP2.6, RCP4.5, RCP6.0 and RCP8.5) in the Two Periods (2050 and 2070), Compared with the Current Potential Distribution.

Compared to the current distribution, in 2050, the total area of the medium suitable region for *P*. *tenuifolia* under the four RCPs (RCP2.6, RCP4.5, RCP6.0 and RCP8.5) will increase by 9.43%, 0.03%, 5.00% and 4.81%, respectively. The total area of the highly suitable district will decrease by 4.09%, 1.00%, 8.37% and 8.55% (Fig 7). Under scenario RCP2.6 2050 and RCP6.0 2050, the areas of the low suitable regions will decline by 1.12% and 0.05%, but almost no change will occur under RCP4.5 2050 and RCP8.5 in 2050. In Northwest Fujian, East Jiangxi, West Zhejiang, Northeast Yunnan, West Guizhou, North Inner Mongolia, East Henan, and East Liaoning, low suitability regions will be converted into medium suitable regions. The region in which medium suitable regions will become low suitable regions is mainly distributed in East Jilin, Hunan, Guizhou, and North Inner Mongolia. The highly suitable region that will be transformed from medium suitable regions includes Southwest Liaoning, North Hebei, Northwest Shanxi, West Henan, Southeast Shaanxi, South Ningxia, and South Gansu. In Central Shandong, Southwest Shanxi, Central Shaanxi, Central Gansu, Central Ningxia, and West Jilin, highly suitable regions will be transformed into medium suitability regions. Altogether, the potential distribution in 2050 will contract in the northeast and expand in southeast, southwest and north (Fig 8).

In 2070, the total areas of the medium suitable region will increase by 9.59%, 0.04%, 1.05% and 2.23%. The total highly suitable habitat areas will decrease by 8.19%, 7.39%, 6.31% and 8.83%. The low suitable regions will increase by 0.64%, 0.39% and 0.43% under RCP4.5, RCP 6.0 and RCP8.5, respectively, but will decline by 0.79% under RCP2.6. The distribution pattern will be similar to that in 2050 and shows an expansion in the north, southeast, and southwest and a contraction in the east, west, and southwest (Fig 8).

**Fig 9:**
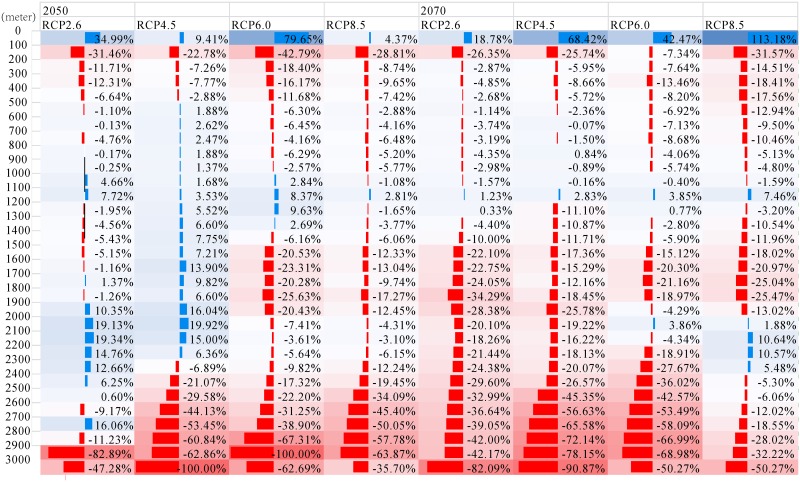
Changes in Every Hundred Meters Elevation of Highly Suitable Habitat for *P*. *tenuifolia* in China. The blue columns indicate the future highly suitable habitat area increased relative to the current in the corresponding altitudinal interval, and the red decreased. The following numbers were the magnitude of changes.

A separate analysis that focused on the altitude patterns among the highly suitable districts (Fig 9) is shown below. Under all scenarios, except for RCP 4.5 2050, the highly suitable habitat area below 100 m will increase in the range of 4.37% to 113.18%, and decrease in the range of -7.34% to -42.79% between 100 m and 200 m. In the range of 200 m to 1500 m, the highly suitable habitat area will fluctuate between -18.41% and 9.63%. The highly suitable habitat area in the range of 1500 m to 2400 m, will vary between -34.29% and 19.92%. Above 2400 m, under all the future scenarios except for RCP2.6 in 2050, highly suitable habitat area will decline from -5.30% to -100% as the increasing elevation.

**Fig 10:**
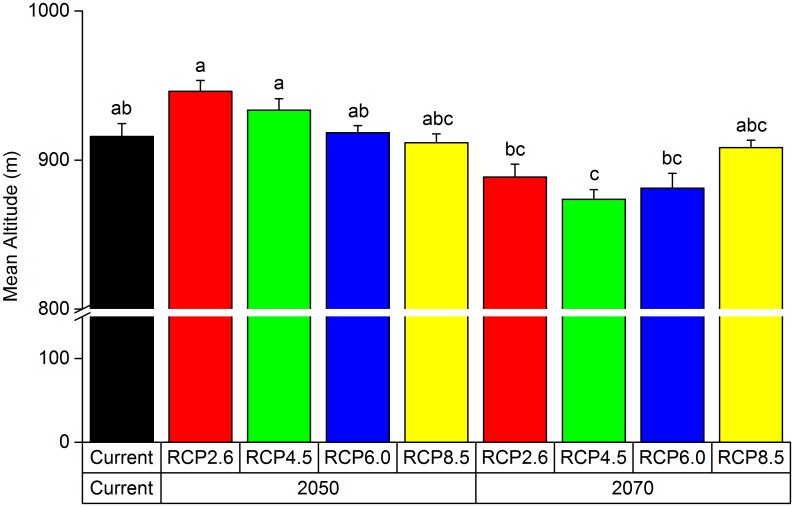
Mean Altitude of the Highly Suitable Regions for *P*. *tenuifolia* in China. Bars indicate means ± sd (n = 10 circle), and significant differences at P < 0.01 within each group are indicated by different letters (a, b and c).

No significant difference was found between the current mean elevation and that predicted in 2050 and 2070. However, a significant difference was found between the mean elevations of highly suitable districts in 2050 and that in 2070 (p < 0.01); the mean elevation of the highly suitable regions in 2050 was higher than that predicted in 2070 (Fig 10).

## Discussion

The rapid increase in the global mean temperature threatens the growth and survival of many wild species[14]. The habitats of many plants have been altered [13], and *P*. *tenuifolia* is no exception. The potential distributions of several traditional Chinese herbs are predicted to change under global warming [29,46,57], and *P*. *tenuifolia* would be impacted as well. Therefore, we modeled the potential distribution of this medicinal plant and predicted how its distribution might be affected under current and eight different future climatic scenarios.

### Prediction Performance of Maxent

In our research, the AUC was adopted to evaluate the discrimination performance of Maxent. The AUC is one of the best model evaluation indexes [48,85]. All of the AUC values for the model predicting the potential distribution of *P*. *tenuifolia* under different climate conditions were above 0.8, which is considered to be very good for Maxent projections of geographic distribution [44,84].

### Dominant environmental variables

The top three variables with high contributions toward determining the distribution of *P*. *tenuifolia* were precipitation in the warmest quarter, annual mean temperature, and altitude. The precipitation in the warmest quarter showed the highest contribution (28.71%) and ranged from 148 mm to 512 mm in highly suitable areas. The annual mean temperature provided an almost identical contribution. The highly suitable range was 8.4°C to 15.4°C. Altitude was a significant variable that could explain the distribution of *P*. *tenuifolia*. Its contribution accounted for 17.13%, and the highly suitable range was 100 m to 2000 m. These results were consistent with the current habit requirements of *P*. *tenuifolia*, which grows well in cool temperatures, is drought resistant, and is commonly found on sunny slopes, forest edges, roadsides, and ridges of fields [7].

### Current suitable habitat

The Maxent predictions showed that the highly suitable habitat of *P*. *tenuifolia* was mainly located in Heilongjiang, Jilin, Liaoning, Inner Mongolia, Hebei, Henan, Beijing, Shanxi, Shaanxi, Ningxia, Gansu, Qinghai, Sichuan, Hubei, Hunan, Shandong, Anhui, Zhejiang and Jiangxi; these regions were consistent with the description in Flora of China [7]. Our research also found 90% of the highly suitable habitat in the current scenario was below 1700 m, and less than 10% of medium suitable habitat located above 2000 m.

### Impacts of climate change on the potential distribution

Previous studies have concluded that global warming will greatly influence species distributions by causing expansions, shifts, or contractions in the species ranges [14,29,86,87]. Our research has revealed that the area of highly suitable habitat for *P*. *tenuifolia* in all eight future scenarios will decrease, and the medium suitable habitat will increase relative to the current area. In four scenarios (RCP2.6 2050, RCP6.0 2050, RCP8.5 2050 and RCP2.6 2070), the low and highly suitable habitat will decline, and the medium suitable habitat will increase. In the other four scenarios (RCP4.5 2050, RCP4.5 2070, RCP6.0 2070 and RCP8.5 2070), the low and medium suitable habitats will increase, and the highly suitable habitat will decline.

Not only the range but also the altitudinal limits of species distribution were affected by global warming. The growth line for plants and butterflies has declined in the Alpine mountain regions[88]. Shrestha found that the Chinese Caterpillar Fungus (*Ophiocordyceps sinensis*) expanded its range at both high and low altitudes [89]. However, in our study, the current mean altitude of highly suitable habitats was lower than that in scenarios RCP2.6 and RCP 4.5 in 2050, almost equal to the value in scenarios RCP6.0 and RCP 8.5 in 2050, but was higher than that in all scenarios in 2070. The centroids of the highly suitable habitat for *P*. *tenuifolia* in all four scenarios in 2050 and RCP2.6 in 2070 moved to lower latitudes, but they moved to higher latitudes in scenarios RCP45, RCP6.0 and RCP8.5 in 2070. Analyzing of the variation in every hundred meters elevation relative to the current suggested that the highly suitable habitat of *P*. *tenuifolia* was easier to be affected by climatic change at high elevation.

The result under different scenarios did not show same tendency, which may be due to several reasons. First, the forecasted ecological niche was wider than the actual niche, which resulted in a larger projected distribution. Second, other environmental variables may affect the distribution range in addition to the selected variables. Third, the MME has been shown to be better than any one model alone [73], but the result has not been validated by observations, and some deviations may occur.

## Conclusions

This study projected the potential distributions of *P*. *tenuifolia* under current and future climate change scenarios and determined the dominant environmental variables that affect changes in the distribution. This result could play an important role in location selection for *P*. *tenuifolia* cultivation and wild *P*. *tenuifolia* reserve design. In addition, the changes in the range and altitude of the highly suitable habitats for *P*. *tenuifolia* were analyzed and compared. The results indicate that the highly suitable habitat of *P*. *tenuifolia* will obviously decrease under future climate change scenarios in 2050 and 2070. We also found that the area of highly suitable habitats below 500 m and above 2300 m will decline in the future. Therefore, the impact of climate change on plant resource protection and sustainable development must be thoroughly investigated, particularly for estimating the extinction risks of environmentally sensitive species.

## Supporting Information

S1 Table Environmental variables used for modeling the potential distribution of *P*. *tenuifolia*. The bold represented the selected variables used to develop the models, and the others were not used to develop the model.
(DOCX)

S2 Table Percent contribution and permutation importance of each environmental variable to model performance under current climatic scenarios in pretest. (DOCX)
