# Supplementary material for: Predicting the Potential Distribution of Polygala tenuifolia Willd. under Climate Change in China
Source: PLoS One. 2016 Sep 23;11(9):e0163718. doi: 10.1371/journal.pone.0163718 (PMC5035090; doi:10.1371/journal.pone.0163718)
Supplement: S1 Table — The bold represented the selected variables used to develop the models, and the others were not used to develop the model. (DOCX) [file pone.0163718.s001.docx]

**S1 Table. Environmental variables used for modeling the potential distribution of *P. tenuifolia.*** The bold represented the selected variables used to develop the models, and the others were not used to develop the model.

| No. | Description | Sources |
| --- | --- | --- |
| **BIO1** | **Annual Mean Temperature** | WorldClim[1] |
| **BIO2** | **Mean Diurnal Range** | WorldClim[1] |
| BIO3 | Isothermality(BIO2/BIO7)(* 100) | WorldClim[1] |
| BIO4 | Temperature Seasonality(Standard Deviation *100) | WorldClim[1] |
| BIO5 | Max Temperature of Warmest Month | WorldClim[1] |
| BIO6 | Min Temperature of Coldest Month | WorldClim[1] |
| BIO7 | Temperature Annual Range(BIO5-BIO6) | WorldClim[1] |
| BIO8 | Mean Temperature of Wettest Quarter | WorldClim[1] |
| BIO9 | Mean Temperature of Driest Quarter | WorldClim[1] |
| BIO10 | Mean Temperature of Warmest Quarter | WorldClim[1] |
| BIO11 | Mean Temperature of Coldest Quarter | WorldClim[1] |
| BIO12 | Annual Precipitation | WorldClim[1] |
| BIO13 | Precipitation of Wettest Month | WorldClim[1] |
| BIO14 | Precipitation of Driest Month | WorldClim[1] |
| **BIO15** | **Precipitation Seasonality** | WorldClim[1] |
| BIO16 | Precipitation of Wettest Quarter | WorldClim[1] |
| BIO17 | Precipitation of Driest Quarter | WorldClim[1] |
| **BIO18** | **Precipitation of Warmest Quarter** | WorldClim[1] |
| **BIO19** | **Precipitation of Coldest Quarter** | WorldClim[1] |
| **ALT** | **Altitude** | CGIAR-CSI[2] |
| **SLOPE** | **Slope** | CGIAR-CSI[2] |
| **ASPECT** | **Aspect** | CGIAR-CSI[2] |
| **LC** | **Land cover** | ISCGM( http://www.iscgm.org/gmd/) |
| **VE** | **Vegetation Coverage** | ISCGM( http://www.iscgm.org/gmd/) |
| T_BS | Topsoil Base Saturation | Harmonized World Soil Database[3] |
| **T_BULK_DENSITY** | **Topsoil Bulk Density** | Harmonized World Soil Database[3] |
| **T_CACO3** | **Topsoil Calcium Carbonate** | Harmonized World Soil Database[3] |
| T_CACO4 | Topsoil Gypsum | Harmonized World Soil Database[3] |
| **T_CEC_CLAY** | **Topsoil CEC (Clay)** | Harmonized World Soil Database[3] |
| T_CEC_SOIL | Topsoil CEC(Soil) | Harmonized World Soil Database[3] |
| T_CLAY | Topsoil Clay Fraction | Harmonized World Soil Database[3] |
| **T_ECE** | **Topsoil Salinity(Elco)** | Harmonized World Soil Database[3] |
| **T_ESP** | **Topsoil Sodicity(ESP)** | Harmonized World Soil Database[3] |
| **T_GRABVEL** | **Topsoil Gravel Content** | Harmonized World Soil Database[3] |
| T_OC | Topsoil Organic Carbon | Harmonized World Soil Database[3] |
| **T_PH_H2O** | **Topsoil pH(H_2_****O)** | Harmonized World Soil Database[3] |
| T_REF_BULK_DENSITY | Topsoil Reference Bulk Density | Harmonized World Soil Database[3] |
| T_SAND | Topsoil Sand Fraction | Harmonized World Soil Database[3] |
| **T_SILT** | **Topsoil Silt Fraction** | Harmonized World Soil Database[3] |
| **T_TEB** | **Topsoil TEB** | Harmonized World Soil Database[3] |
| T_TEXTURE | Topsoil Texture | Harmonized World Soil Database[3] |
| **T_USDA_TEX_CLASS** | **Topsoil USDA Texture Classification** | Harmonized World Soil Database[3] |

1. Hijmans RJ, Cameron SE, Parra JL, Jones PG, Jarvis A. Very high resolution interpolated climate surfaces for global land areas. International Journal of Climatology. 2005;25: 1965-1978. doi: 10.1002/joc.1276

2. Jarvis A, Reuter HI, Nelson A, Guevara E. Hole-filled SRTM for the globe Version. 2008. Available: the CGIAR-CSI SRTM 90m Database (http://srtm.csi.cgiar.org).

3. Nachtergaele. F, Velthuizen. Hv, Verelst. L, Wiberg. D, Batjes. N, Dijkshoorn. K, et al. Harmonized world soil database (Version 1.2). Laxenburg, Austria: Food and Agriculture Organization of the UN, International Institute for Applied Systems Analysis, ISRIC - World Soil Information, Institute of Soil Science - Chinese Academy of Sciences, Joint Research Centre of the EC; 2012.
