# Supplementary material for: Predicting the Potential Distribution of Polygala tenuifolia Willd. under Climate Change in China
Source: PLoS One. 2016 Sep 23;11(9):e0163718. doi: 10.1371/journal.pone.0163718 (PMC5035090; doi:10.1371/journal.pone.0163718)
Supplement: S2 Table — (DOCX) [file pone.0163718.s002.docx]

**S2 Table. Percent contribution and permutation importance of each environmental variable to model performance under current climatic scenarios in pretest.**

| Variable | Percent contribution | Permutation importance |
| --- | --- | --- |
| BIO1 | 30.6 | 8.3 |
| BIO18 | 16.2 | 5.6 |
| BIO9 | 12.7 | 0.1 |
| BIO19 | 7.7 | 3.4 |
| BIO13 | 7 | 13.1 |
| BIO8 | 6.1 | 10.1 |
| BIO3 | 4.3 | 2.3 |
| BIO4 | 3.8 | 1.4 |
| T_GRAVEL | 2.3 | 11.8 |
| SLPOE | 2.1 | 5.1 |
| BIO12 | 1.2 | 1.4 |
| BIO11 | 0.8 | 4.4 |
| BIO15 | 0.8 | 11 |
| BIO7 | 0.8 | 1.3 |
| BIO6 | 0.7 | 5.9 |
| ALT | 0.4 | 2.7 |
| BIO10 | 0.4 | 3.1 |
| T_USDA_TEX_CLASS | 0.3 | 0.4 |
| T_PH_H2O | 0.3 | 2.1 |
| BIO14 | 0.2 | 0.2 |
| BIO2 | 0.2 | 1 |
| LC | 0.2 | 0.8 |
| ASPECT | 0.2 | 0.3 |
| T_BULK_DENSITY | 0.1 | 0.2 |
| T_ESP | 0.1 | 0.5 |
| T_TEB | 0.1 | 0.2 |
| BIO5 | 0.1 | 0.5 |
| T_CEC_CLAY | 0.1 | 0.3 |
| T_REF_BULK_DENSITY | 0.1 | 0.4 |
| T_SAND | 0 | 0.4 |
| T_SILT | 0 | 0.9 |
| VE | 0 | 0.2 |
| T_BS | 0 | 0.1 |
| BIO17 | 0 | 0.4 |
| T_CACO3 | 0 | 0 |
| T_TEXTURE | 0 | 0 |
| T_CLAY | 0 | 0.1 |
| BIO16 | 0 | 0 |
| T_CEC_SOIL | 0 | 0 |
| T_ECE | 0 | 0 |
| T_CASO4 | 0 | 0 |
| T_OC | 0 | 0 |
